# Supplementary material for: Between and Within-Country Variations in Infant and Young Child Feeding Practices in South Asia
Source: Int J Environ Res Public Health. 2022 Apr 5;19(7):4350. doi: 10.3390/ijerph19074350 (PMC8998566; doi:10.3390/ijerph19074350)
Supplement: Supplementary file 1 [file ijerph-19-04350-s001.zip › ijerph-1584092-SI.pdf]

International Journal of
Environmental Research and Public Health

Table S1. Description of WHO eight-core IYCF indicators.

Indicators	Definition	Calculation
1. Early initiation of breastfeeding	The proportion of children born in the last 24 months who were put to the breast within one hour of birth	(Children born in the last 24 months who were put to the breast within one hour of birth) ÷ (Children born in the last 24 months)
2. Exclusive breastfeeding under 6 months	The proportion of infants 0–5 months of age who are fed exclusively with breast milk	(Infants 0–5 months of age who received only breast milk during the previous day) ÷ (Infants 0–5 months of age)
3. Continued breastfeeding at 1 year	The proportion of children 12–15 months of age who are fed breast milk	(Children 12–15 months of age who received breast milk during the previous day) ÷ (Children 12–15 months of age)
4. Introduction of solid, semi-solid, or soft foods	The proportion of infants 6–8 months of age who receive solid, semi-solid, or soft foods	(Infants 6–8 months of age who received solid, semi-solid or soft foods during the previous day) ÷ (Infants 6–8 months of age)
5. Minimum dietary diversity	The proportion of children 6–23 months of age who receive foods from 4 or more food groups	Children 6–23 months of age who received foods from ≥ 4 food groups during the previous day Children 6–23 months of age
6. Minimum meal frequency	The proportion of breastfed and non-breastfed children 6–23 months of age who receive solid, semi-solid, or soft foods (but also including milk feeds for non-breastfed children) the minimum number of times or more	(Breastfed children 6–23 months of age who received solid, semi-solid or soft foods the minimum number of times or more during the previous day) ÷ (Breastfed children 6–23 months of age)<br>And<br>(Non-breastfed children 6–23 months of age who received solid, semi-solid or soft foods or milk feeds the minimum number of times or more during the previous day) ÷ (Non-breastfed children 6–23 months of age)
7. Minimum acceptable diet	The proportion of children 6–23 months of age who receive a minimum acceptable diet (apart from breast milk).	(Breastfed children 6–23 months of age who had at least the minimum dietary diversity and the minimum meal frequency during the previous day) ÷ (Breastfed children 6–23 months of age)<br>And<br>(Non-breastfed children 6–23 months of age who received at least 2 milk feedings and had at least the minimum dietary diversity not including milk feeds and the minimum meal frequency during the previous day) ÷ (Non-breastfed children 6–23 months of age)
8. Consumption of iron-rich or iron-fortified foods	The proportion of children 6–23 months of age who receive an iron-rich food or iron-fortified food that is specially designed for infants and young children or that is fortified in the home.	(Children 6–23 months of age who received an iron-rich food or food that was specially designed for infants and young children and was fortified with iron, or a food that was fortified in the home with a product that included iron during the previous day) ÷ (Children 6–23 months of age)

Table S2: Prevalence of IYCF indicators across mother's age

Indicators	Pooled			Afghanistan			Bangladesh			India			Maldives			Nepal			Pakistan		
	**15–24**	**25–34**	**≥35**	**15–24**	**25–34**	**≥35**	**15–24**	**25–34**	**≥35**	**15–24**	**25–34**	**≥35**	**15–24**	**25–34**	**≥35**	**15–24**	**25–34**	**≥35**	**15–24**	**25–34**	**≥35**
EIBF (%)	44.7	45.7	47.0	41.8	41.8	43.7	57.9	63.3	65.6	43.7	43.6	39.8	65.2	69.6	66.8	57.6	53.3	54.3	19.6	20.4	25.1
	p <0.001			p = 0.631			p = 0.013			p = 0.002			p = 0.620			p = 0.241			p = 0.162		
EBF (%)	55.6	52.7	49.1	43.6	42.7	35.9	67.7	62.4	44.3	54.6	51.2	48.2	52.3	69.3	54.5	67.7	62.8	88.1	49.7	43.6	42.2
	p <0.001			p = 0.021			p <0.001			p <0.001			0.175			p = 0.176			p = 0.683		
CBF (%)	82.7	83.3	80.1	71.3	76.9	83.1	91.9	93.8	93.0	83.3	83.6	78.8	76.3	76.1	75.5	94.1	97.4	74.4	66.1	66.3	72.9
	p = 0.006			p = 0.027			p = 0.731			p = 0.030			p = 0.997			p = 0.002			p = 0.642		
ISSSF (%)	48.8	50.8	54.6	61.0	56.0	56.8	66.6	69.7	97.8	44.6	44.0	39.2	90.1	82.0	73.5	75.5	79.4	90.4	56.1	60.2	43.2
	p <0.001			p = 0.586			p = 0.045			p = 0.206			p= 0.367			p = 0.463			p = 0.169		
MDD (%)	20.3	23.1	22.6	23.2	25.1	17.6	38.3	36.9	37.2	19.4	22.1	19.2	64.0	74.4	69.3	42.2	48.8	35.7	18.5	19.3	15.7
	p <0.001			p = 0.008			p = 0.839			p <0.001			p = 0.173			p = 0.028			p = 0.480		
MMF (%)	39.1	40.4	41.5	49.4	49.4	47.5	79.1	78.0	85.9	34.3	35.3	31.8	71.7	64.3	62.6	70.5	69.3	71.0	58.0	59.3	51.7
	p <0.001			p = 0.763			p = 0.163			p = 0.003			p = 0.306			p = 0.892			p = 0.137		
MAD (%)	11.9	13.4	13.5	16.7	17.7	13.3	35.2	34.1	36.4	10.1	11.4	9.0	49.7	53.0	51.5	31.5	38.1	28.6	17.1	16.8	13.6
	p <0.001			p = 0.078			p = 0.819			p <0.001			p = 0.848			p = 0.410			p = 0.453		
CIRF (%)	19.7	21.9	26.3	31.8	31.8	23.8	69.0	66.9	65.9	16.7	17.5	15.8	62.0	70.0	75.1	35.3	34.7	25.3	35.1	38.9	33.5
	p <0.001			p = 0.001			p = 0.567			p = 0.094			p = 0.153			p = 0.475			p = 0.259		

EIBF=Early initiation of breastfeeding, EBF= Exclusive breastfeeding, CBF=Continued breastfeeding at 1 year, ISSSF=Introduction of solid semi-solid and soft foods, MDD=Minimum dietary diversity, MMF=Minimum meal frequency, MAD=Minimum acceptable diet, CIRF=Consumption of iron-rich or iron-fortified foods.
